# Supplementary material for: Minimum entropy production by microswimmers with internal dissipation
Source: Nat Commun. 2023 Sep 28;14:6060. doi: 10.1038/s41467-023-41280-z (PMC10539332; doi:10.1038/s41467-023-41280-z)
Supplement: Supplementary file 1 — Supplementary Information [file 41467_2023_41280_MOESM1_ESM.pdf]

# Supplementary Information for "Minimum Entropy Production by Microswimmers with Internal Dissipation"


Abdallah Daddi-Moussa-Ider,[1] Ramin Golestanian,[1,2] and Andrej Vilfan[1,3,*]

[1]*Max Planck Institute for Dynamics and Self-Organization (MPI-DS), 37077 Göttingen, Germany*
[2]*Rudolf Peierls Centre for Theoretical Physics, University of Oxford, Oxford OX1 3PU, United Kingdom*
[3]*Jožef Stefan Institute, 1000 Ljubljana, Slovenia*



(Dated: August 20, 2023)


## SUPPLEMENTARY NOTE 1: PROOF OF GENERALIZED PASSIVE MINIMUM DISSIPATION THEOREMS

The Helmholtz minimum dissipation theorem states that among all incompressible flows that satisfy a prescribed fixed-velocity boundary condition, the Stokes flow has the minimum dissipation. In other words, any perturbation to the Stokes flow that satisfies the same boundary condition leads to an increase in the total dissipation. In the following, we generalize the minimum dissipation theorem to two configurations. The first generalization allows the presence of fluid-fluid interfaces (Eq. (2) in the main text) and the second includes surfaces with the Navier-slip boundary condition (Eq. (5) in the main text). Our derivation is adapted from the proof of the original theorem as formulated in the textbook by Guazzelli and Morris [1].

**Fluid-fluid interface.** We first prove the statement that among all flows around a fluid-fluid interface of a fixed shape, the flow without tangential stress on the surface has the minimum dissipation. We label the exterior volume as $\mathcal{V}$ and the interior as $\mathcal{V}_\mathrm{i}$. The solution in both domains with zero tangential traction at the interface is described with the velocity field $\mathbf{v}$. The total dissipation in both domains is determined as

$$P = \left[2\mu \int_{\mathcal{V}} + 2\mu_\mathrm{i} \int_{\mathcal{V}_\mathrm{i}}\right] \mathrm{d}V \, \mathbf{E} : \mathbf{E} \, , \tag{1}$$

with $\mathbf{E}$ denoting the rate-of-strain tensor and $\mathbf{E} : \mathbf{E} = \sum_{\alpha,\beta} E_{\alpha\beta} E_{\alpha\beta}$ the Frobenius inner product. The expression in brackets is used as a shortcut for the sum of multiple integrals with the same integrand. We now introduce a perturbation $\mathbf{v}'$ that satisfies the condition $\mathbf{v}' \cdot \mathbf{n} = 0$ at the interface. The corresponding perturbation of the strain rate is $\mathbf{E}' = \frac{1}{2}\left(\boldsymbol{\nabla}\mathbf{v}' + \boldsymbol{\nabla}\mathbf{v}'^{\top}\right)$. The perturbation alters the dissipation by

$$\begin{aligned} \Delta P &= \left[2\mu \int_{\mathcal{V}} + 2\mu_\mathrm{i} \int_{\mathcal{V}_\mathrm{i}}\right] \mathrm{d}V \left[(\mathbf{E}' + \mathbf{E}) : (\mathbf{E}' + \mathbf{E}) - \mathbf{E} : \mathbf{E}\right] \\ &= \left[2\mu \int_{\mathcal{V}} + 2\mu_\mathrm{i} \int_{\mathcal{V}_\mathrm{i}}\right] \mathrm{d}V \, \mathbf{E}' : \mathbf{E}' + \left[4\mu \int_{\mathcal{V}} + 4\mu_\mathrm{i} \int_{\mathcal{V}_\mathrm{i}}\right] \mathrm{d}V \, \mathbf{E}' : \mathbf{E} \, . \end{aligned} \tag{2}$$

We first show that the second term vanishes if the flow $\mathbf{v}$ satisfies the continuity of tangential stress at the interface. Since both $\mathbf{E}'$ and $\mathbf{E}$ are traceless symmetric tensors, and $\boldsymbol{\nabla} \cdot \mathbf{v}' = 0$, we have $2\mu \mathbf{E}' : \mathbf{E} = \boldsymbol{\nabla}\mathbf{v}' : \boldsymbol{\sigma}$, where $\boldsymbol{\sigma} = -p\mathbf{I} + 2\mu\mathbf{E}$ is the stress field for the unperturbed flow and $p$ is the corresponding pressure field. Using $\boldsymbol{\nabla} \cdot \boldsymbol{\sigma} = \mathbf{0}$, one obtains $\boldsymbol{\nabla}\mathbf{v}' : \boldsymbol{\sigma} = \boldsymbol{\nabla} \cdot (\mathbf{v}' \cdot \boldsymbol{\sigma})$. Thence, by using the divergence theorem, we find $4\mu \int_{\mathcal{V}} \mathrm{d}V \, \mathbf{E}' : \mathbf{E} = -2 \int_{\mathcal{S}} \mathrm{d}S \, \mathbf{n} \cdot \boldsymbol{\sigma} \cdot \mathbf{v}'$ and analogously for the internal domain $4\mu_\mathrm{i} \int_{\mathcal{V}_\mathrm{i}} \mathrm{d}V \, \mathbf{E}' : \mathbf{E} = 2 \int_{\mathcal{S}} \mathrm{d}S \, \mathbf{n} \cdot \boldsymbol{\sigma}_\mathrm{i} \cdot \mathbf{v}'$. The sum of the two terms vanishes at the interface, because $(\mathbf{I} - \mathbf{nn}) \cdot (\boldsymbol{\sigma} - \boldsymbol{\sigma}_\mathrm{i}) \cdot \mathbf{n} = \mathbf{0}$ and $\mathbf{n} \cdot \mathbf{v}' = 0$.

Now, noting that the first term in Eq. (2), $\int \mathrm{d}V \, \mathbf{E}' : \mathbf{E}'$, is positive-definite, we can conclude that the minimum dissipated power can be only achieved when $\mathbf{E}' = \mathbf{0}$, thereby indicating that the flow that minimizes dissipation is that with vanishing tangential traction at the interface. This way we have proven that the dissipation of any flow around an interface with velocity continuity, itself moving with velocity $\mathbf{V}$, satisfies the inequality

$$P \geq \mathbf{V} \cdot \mathbf{R}_\mathrm{Droplet} \cdot \mathbf{V} \, , \tag{3}$$

which is Eq. (2) in the main text.


* andrej.vilfan@ds.mpg.de

**Surface with Navier slip.** In the second case, we consider the dissipation in the surrounding fluid together with surface dissipation as stated in Eq. (3) in the main text. A flow perturbation $\mathbf{v}'$ changes the total dissipation by

$$\Delta P = 2\mu \int_{\mathcal{V}} \mathrm{d}V\, \mathbf{E}' : \mathbf{E}' + 4\mu \int_{\mathcal{V}} \mathrm{d}V\, \mathbf{E}' : \mathbf{E} + \frac{\mu}{\lambda} \int_{\mathcal{S}} \mathrm{d}S\, \mathbf{v}'^2 + 2\, \frac{\mu}{\lambda} \int_{\mathcal{S}} \mathrm{d}S\, \tilde{\mathbf{v}} \cdot \mathbf{v}' \,. \tag{4}$$

Here, the first two terms represent the change of external dissipation given by Eq. (2) and the last two the change of internal dissipation. Again, the first and the third term are positive for any non-vanishing $\mathbf{v}'$. The second term evaluates to $-2 \int_{\mathcal{S}} \mathrm{d}S\, \mathbf{f} \cdot \mathbf{v}'$. Together with the fourth term, it yields 0 when $\mathbf{f}^{\parallel} = (\mu/\lambda)\, \tilde{\mathbf{v}}$ at the surface. We have thus demonstrated that the combined external and surface dissipation of a flow (Eq. (3) in the main text) is minimal if the flow satisfies the Navier-slip condition with slip length $\lambda$. The inequality for the dissipation in any flow around that body moving with velocity $\mathsf{V}$ is

$$P \geq \mathsf{V} \cdot \mathsf{R}_{\mathrm{Navier}} \cdot \mathsf{V} \,, \tag{5}$$

proving Eq. (5) from the main text. $\mathsf{R}_{\mathrm{Navier}}$ denotes the generalized drag coefficient of the rigid body with shape $\mathcal{S}$ and slip length $\lambda$.

## SUPPLEMENTARY NOTE 2: DISSIPATION RATE

Here we recapitulate the relationships for the total dissipation in the flow around active or passive bodies in an otherwise quiescent Stokes fluid (e.g., Ref. [1]). The total dissipation rate in a fluid can be expressed as the integral of the dissipation density

$$P = 2\mu \int_{\mathcal{V}} \mathrm{d}V\, \mathbf{E} : \mathbf{E} \,. \tag{6}$$

Because of energy conservation, the dissipation rate has to be equal to the flux of work from the object to the fluid. This can be shown by applying the divergence theorem to Eq. (6) and obtaining

$$P = -\int_{\mathcal{S}} \mathrm{d}S\, \mathbf{f} \cdot \mathbf{v} \,. \tag{7}$$

Here $\mathbf{f} = \boldsymbol{\sigma} \cdot \mathbf{n}$ denotes the traction on the surface of the particle. If the particle exerts forces on the fluid anywhere else outside its surface, they can be included in the form of a volume integral $-\int_{\mathcal{V}} \mathrm{d}V \mathfrak{f} \cdot \mathbf{v}$. We can also express the velocity field $\mathbf{v}$ in terms of the velocity field in the co-moving frame $\tilde{\mathbf{v}}$ and the rigid-body velocity $\mathsf{V} = [\mathbf{V}, \boldsymbol{\Omega}]$ as $\mathbf{v} = \tilde{\mathbf{v}} + \mathbf{V} + \boldsymbol{\Omega} \times \mathbf{x}$. Then the dissipated power can be decomposed into the work of the active forces on the body and the work of the external forces:

$$P = -\int_{\mathcal{S}} \mathrm{d}S\, \mathbf{f} \cdot \tilde{\mathbf{v}} - \mathsf{F} \cdot \mathsf{V} \,. \tag{8}$$

Here $\mathsf{F} = [\mathbf{F}, \mathbf{M}]$ is the generalized force on the body with $\mathbf{F} = \int_{\mathcal{S}} \mathrm{d}S\, \mathbf{f}$ and $\mathbf{M} = \int_{\mathcal{S}} \mathrm{d}S\, \mathbf{x} \times \mathbf{f}$.

For a passive body, the force $\mathbf{F}$ and torque $\mathbf{M}$ on the body are linear functions of the translational and rotational velocities and can therefore be expressed with a generalized drag coefficient $\mathsf{R}$ as $\mathsf{F} = -\mathsf{R} \cdot \mathsf{V}$, or with components:

$$\begin{bmatrix} \mathbf{F} \\ \mathbf{M} \end{bmatrix} = \begin{bmatrix} \mathbf{R}_{\mathrm{TT}} & \mathbf{R}_{\mathrm{RT}}^{\top} \\ \mathbf{R}_{\mathrm{RT}} & \mathbf{R}_{\mathrm{RR}} \end{bmatrix} \cdot \begin{bmatrix} \mathbf{V} \\ \boldsymbol{\Omega} \end{bmatrix} . \tag{9}$$

The rate of work by the external force is $\mathsf{V} \cdot \mathsf{R} \cdot \mathsf{V}$. According to Eq. (8), it equals the dissipation in the fluid plus the dissipation in the boundary $\mathbf{f} \cdot \tilde{\mathbf{v}}$ if the latter is not zero (for the Navier slip boundary).

## SUPPLEMENTARY NOTE 3: DRAG COEFFICIENTS OF COMPOSITE PASSIVE BODIES WITH SPHERICAL SHAPE

We determine the viscous flow field past composite passive bodies of spherical geometry. The body consists of an outer shell with the radius $a$ and an inner core with the radius $b$. Following the notation used in the main body of the paper, we use the subscript $i$ for the flow variables in the interior fluid domain bounded by the inner and outer

spherical surfaces, such that $b \le r \le a$. Absence of subscript indicates the flow variables in the exterior fluid domain, such that $r \ge a$. Moreover, we assume that the dynamic viscosity is the same everywhere in the fluid medium. We formulate the solution of the Stokes equation in the reference frame attached to the particle. The solution for the stream function satisfying the regularity condition at infinity can be expressed in spherical coordinates as [2]

$$\psi_{\mathrm{i}}(r,\theta) = b^2 V \left( A\,\frac{r}{b} + B \left(\frac{r}{b}\right)^2 + C \left(\frac{r}{b}\right)^4 + D\,\frac{b}{r} \right) \sin^2\theta \,, \tag{10}$$

$$\psi(r,\theta) = b^2 V \left( E\,\frac{r}{b} + G \left(\frac{r}{b}\right)^2 + H\,\frac{b}{r} \right) \sin^2\theta \,, \tag{11}$$

where $A$, $B$, $C$, $D$, $E$, $G$, and $H$ are constants to be determined from the boundary conditions prescribed at the inner/outer radius or at infinity. The corresponding hydrodynamic pressure field reads

$$p_{\mathrm{i}}(r,\theta) = -\frac{2\mu V}{b} \left( A \left(\frac{b}{r}\right)^2 + 10C\,\frac{r}{b} \right) \cos\theta \,, \tag{12}$$

$$p(r,\theta) = -\frac{2\mu V}{b}\, E \left(\frac{b}{r}\right)^2 \cos\theta \,. \tag{13}$$

In the limit $r \to \infty$, the stream function has to converge to that of a uniform flow with the velocity $-V\hat{\mathbf{e}}_z$, $\psi_2 = \frac{V}{2}\, r^2 \sin^2\theta$ [2]. Accordingly, we obtain $G = 1/2$.

**Composite body with zero tangential velocity.** We determine in the following the flow around a passive body consisting of an inner sphere with a no-slip boundary and an outer sphere which imposes zero tangential velocity and zero normal traction, as shown in Supplementary Figure 1a. At $r = b$, we set $\mathbf{v} = \mathbf{0}$. In addition, we require at $r = a$ that $v_\theta = 0$ and assume that both $\mathbf{v}$ and $\sigma_{rr}$ are continuous. Defining $\alpha = b/a \in [0, 1]$, we obtain

$$\begin{aligned}
A &= -2\beta\alpha^3 \left(6 + 6\alpha + \alpha^2 + \alpha^3 + \alpha^4\right) \,, \\
B &= \beta\alpha^3 \left(8 + 8\alpha + 8\alpha^2 + 3\alpha^3 + 3\alpha^4\right) \,, \\
C &= -\beta\alpha^5 \left(4 + \alpha + \alpha^2\right) \,, \\
D &= 2\beta\alpha^3 \left(2 + 2\alpha - \alpha^2\right) \,, \\
E &= -2\beta\alpha^2 \left(20 + 11\alpha + 11\alpha^2 + \alpha^3 + \alpha^4 + \alpha^5\right) \,, \\
H &= 2\beta \left(8 + 2\alpha + 2\alpha^2 + 2\alpha^3 + 2\alpha^4 - \alpha^5\right) \,,
\end{aligned} \tag{14}$$

where we have defined the abbreviation $\beta^{-1} = 2\alpha^3 \left(28 + 13\alpha + 13\alpha^2 + 3\alpha^3 + 3\alpha^4\right)$.

The hydrodynamic drag force exerted on the composite can be determined from the monopole term as $F = 8\pi\mu b E$ [2]. Then, the drag coefficient $R_{\mathrm{CP}} \equiv -F/V$ can be expressed in a scaled form as

$$\frac{R_{\mathrm{CP}}}{6\pi\mu a} = \frac{4}{3} \cdot \frac{20 + 11\alpha + 11\alpha^2 + \alpha^3 + \alpha^4 + \alpha^5}{28 + 13\alpha + 13\alpha^2 + 3\alpha^3 + 3\alpha^4} \in \left[\frac{20}{21}, 1\right] \,. \tag{15}$$

In particular, by setting $\alpha = 1 - \epsilon$, it follows that $R_{\mathrm{CP}} = 6\pi\mu a \left(1 - \frac{1}{12}\,\epsilon^3 + \mathcal{O}\left(\epsilon^4\right)\right)$.

**Droplet with a no-slip core.** The second passive body consists of a no-slip inner sphere surrounded by a fluid-fluid interface with equal viscosities on both sides, which imposes zero normal velocity, zero tangential traction and the continuity of tangential velocity (Supplementary Figure 1b). At $r = b$, we impose $\mathbf{v} = \mathbf{0}$. We require that $v_r = 0$ at $r = a$ and that both $\mathbf{v}$ and $\sigma_{r\theta}$ are continuous at $r = a$. We obtain

$$\begin{aligned}
A &= \beta\alpha^3 \left(3 + 6\alpha + 4\alpha^2 + 2\alpha^3\right) \,, \\
B &= -\beta\alpha^3 \left(2 + 4\alpha + 6\alpha^2 + 3\alpha^3\right) \,, \\
C &= \beta\alpha^5 \left(2 + \alpha\right) \,, \\
D &= -\beta\alpha^3 \left(1 + 2\alpha\right) \,, \\
E &= -2\beta\alpha^2 \left(1 - \alpha\right) \left(5 + 6\alpha + 3\alpha^2 + \alpha^3\right) \,, \\
H &= \beta \left(1 - \alpha^2\right) \left(2 + \alpha + 2\alpha^2\right) \,,
\end{aligned} \tag{16}$$

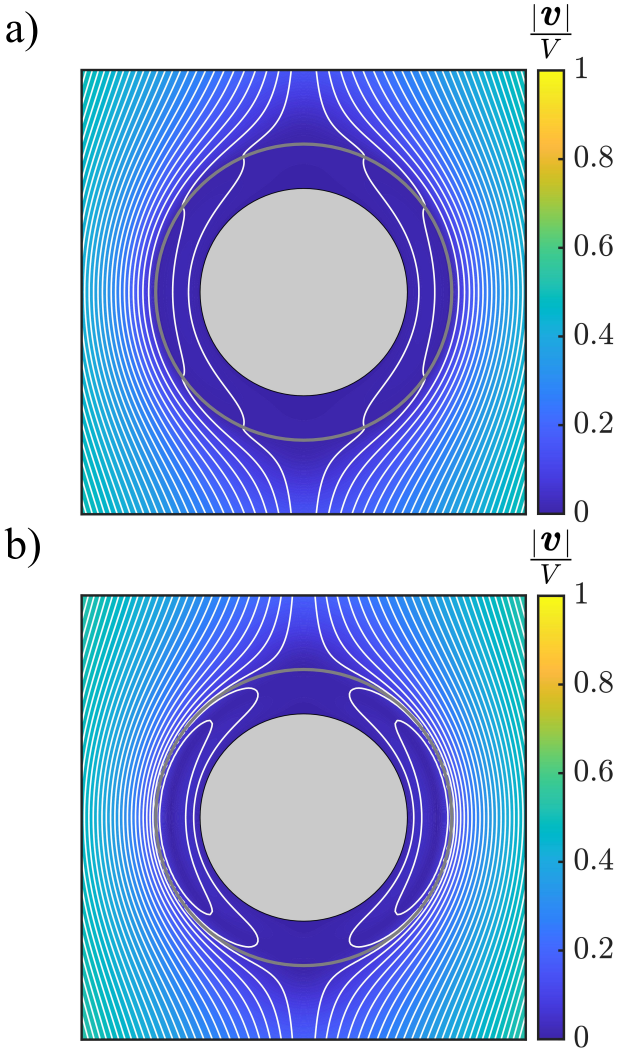


**Supplementary Figure 1. Streamlines and velocities of the flow around two composite passive bodies.** a) Body with a no-slip boundary condition at the inner sphere and zero tangential velocity at the outer sphere. b) Body with a no-slip inner core and a non-deformable fluid-fluid interface at the outer surface.

wherein $\beta^{-1} = 2\alpha^3 (1-\alpha)\left(8 + 9\alpha + 3\alpha^2\right)$. The corresponding drag coefficient is obtained as

$$\frac{R_{\mathrm{DC}}}{6\pi\mu a} = \frac{4}{3} \cdot \frac{5 + 6\alpha + 3\alpha^2 + \alpha^3}{8 + 9\alpha + 3\alpha^2} \in \left[\frac{5}{6}, 1\right] . \tag{17}$$

For $\alpha = 1 - \epsilon$, we obtain $R_{\mathrm{DC}} = 6\pi\mu a \left(1 - \frac{1}{4}\epsilon + \mathcal{O}\left(\epsilon^2\right)\right)$.

## SUPPLEMENTARY NOTE 4: RESPONSE OF THE SWIMMER TO AN EXTERNAL FORCE

In the following we derive the expression for the power expenditure by an active swimmer when it is subject to an additional external force (Eq. (35) in the main text). The assumption is that the active forces produced by the swimmer remain unaffected, but all flow velocities change in response to the applied force. The flow of the active swimmer takes the form

$$\mathbf{v}_{\mathrm{A}} = \mathbf{v}_{\mathrm{A}}^{0} + \mathbf{v}_{\mathrm{V}}' \tag{18}$$

where $\mathbf{v}_{\mathrm{V}}'$ is the flow of the solution of the V-problem (Droplet, No-slip, etc.) when pulled by the force $\mathbf{F}_{\mathrm{ext}}$. The rate of dissipation in the fluid evaluates to (note that the external force has the opposite sign from the drag force in

Eq. (8))

$$P_{\rm A} = P_{\rm A}^0 - \int {\rm d}S \mathbf{f}_{\rm A}^0 \cdot \tilde{\mathbf{v}}_{\rm V}' + \mathbf{F}_{\rm ext} \cdot \mathbf{V}_{\rm A} \ . \tag{19}$$

Now we apply the Lorentz reciprocal theorem as formulated in Eq. (46) in the main text and obtain

$$P_{\rm A} = P_{\rm A}^0 - \int {\rm d}S \mathbf{f}_{\rm V}' \cdot \tilde{\mathbf{v}}_{\rm A}^0 + \mathbf{F}_{\rm A}^0 \cdot \mathbf{V}_{\rm V}' - \mathbf{F}_{\rm V}' \cdot \mathbf{V}_{\rm A}^0 + \mathbf{F}_{\rm ext} \cdot \mathbf{V}_{\rm A} \ . \tag{20}$$

The product in the integral is zero in all problems studied except for the surface dissipation, for which the assumption of a constant force density needs to be adapted. In addition, $\mathbf{F}_{\rm A}^0 = 0$ for the unperturbed active swimmer, and the drag on the passive body is opposite equal to the external force acting on it, $\mathbf{F}_{\rm V}' = -\mathbf{F}_{\rm ext}$. We therefore obtain

$$P_{\rm A} = P_{\rm A}^0 + \mathbf{F}_{\rm ext} \cdot (\mathbf{V}_{\rm A}^0 + \mathbf{V}_{\rm A}) \, , \tag{21}$$

which is Eq. (35) from the main text.


[1] E. Guazzelli and J. F. Morris, *A Physical Introduction to Suspension Dynamics* (Cambridge University Press, 2009).
[2] J. Happel and H. Brenner, *Low Reynolds Number Hydrodynamics* (Springer Netherlands, 1983).
